# Management of liver metastases from uveal melanoma

**DOI:** 10.1093/bjs/znaf130

**Published:** 2025-08-05

**Authors:** Anne Huibers, Andrew Wong, Mark Burgmans, Lars Ny, Gustav Stålhammar, Ellen Kapiteijn, Jonathan S Zager, Roger Olofsson Bagge

**Affiliations:** Department of Surgery, Sahlgrenska University Hospital, Gothenburg, Sweden; Department of Surgery, Institute of Clinical Sciences, Sahlgrenska Academy, University of Gothenburg, Gothenburg, Sweden; Department of Surgery, Institute of Clinical Sciences, Sahlgrenska Academy, University of Gothenburg, Gothenburg, Sweden; Department of Radiology, Sahlgrenska University Hospital, Gothenburg, Sweden; Department of Radiology, Leiden University Medical Centre, Leiden, The Netherlands; Department of Oncology, Institute of Clinical Sciences, Sahlgrenska Academy, University of Gothenburg, Gothenburg, Sweden; Department of Oncology, Sahlgrenska University Hospital, Gothenburg, Sweden; Department of Clinical Neuroscience, Division of Eye and Vision, St. Erik Eye Hospital, Karolinska Institutet, Stockholm, Sweden; Department of Medical Oncology, Leiden University Medical Centre, Leiden, The Netherlands; Department of Cutaneous Oncology, Moffitt Cancer Center, Tampa, Florida, USA; Department of Oncologic Sciences, University of South Florida Morsani College of Medicine, Tampa, Florida, USA; Department of Surgery, Sahlgrenska University Hospital, Gothenburg, Sweden; Department of Surgery, Institute of Clinical Sciences, Sahlgrenska Academy, University of Gothenburg, Gothenburg, Sweden

## Abstract

Uveal melanoma is the most common primary intraocular malignancy in adults, with distinct genetic and clinical characteristics compared with cutaneous melanoma. Despite improvements in the treatment of the primary tumour, nearly half of the patients will develop distant metastases, most commonly in the liver. Once metastases are detected, the median overall survival is approximately 1 year, with a 2-year survival rate of only 8%. Systemic treatment, including chemotherapy, immunotherapy, and targeted therapy, has historically shown limited efficacy. The first (and so far only) systemic treatment to demonstrate an improvement in overall survival is tebentafusp, which is now approved for treatment of patients with metastatic or unresectable uveal melanoma and an HLA-A*02:01 genotype. Liver-directed therapies include surgical resection, radioembolization, chemoembolization, immune-embolization, isolated hepatic perfusion, and percutaneous hepatic perfusion. This review discusses the clinical background of uveal melanoma and liver metastasis, the efficacy of systemic and locoregional treatment options, and the promising development of combining locoregional liver-directed treatment with systemic treatment.

## Teaser

This review discusses the clinical background of uveal melanoma and liver metastasis, the efficacy of systemic and locoregional treatment options, and the development of combining locoregional liver-directed treatment with systemic treatment.

## Introduction

**Fig. 1:**
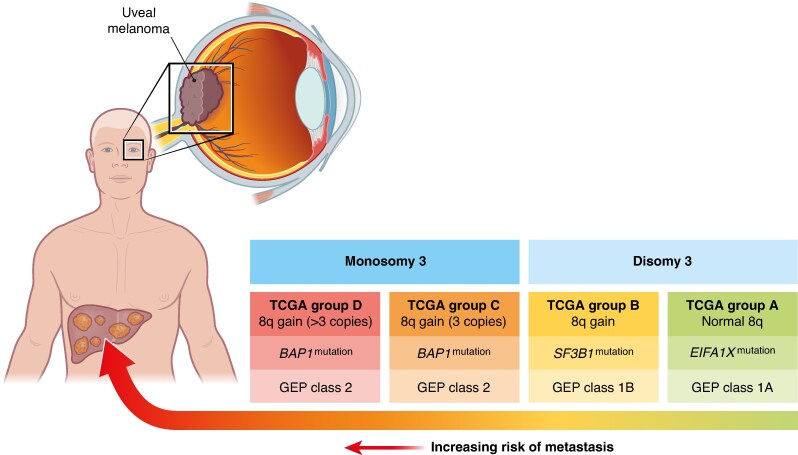
Genetic and cytogenetic determinants of metastatic risk in uveal melanoma GEP, gene expression profiling.

Uveal melanoma is the most common primary intraocular malignancy in adults and represents the most common non-cutaneous melanoma^1^. It arises from melanocytes within the uveal tract, a layer beneath the sclera of the eye, including the iris, ciliary body, and choroid. Choroidal melanomas are the predominant subtype, accounting for nearly 90% of patients, followed by the ciliary body (6%) and the iris (4%)^1^ (*Fig. 1*). The incidence of uveal melanoma has increased over the past several decades, rising from approximately 8 new cases per million inhabitants per year in Northern Europe in the 1960s to 12 new cases per million inhabitants per year by 2022^2–6^. The incidence rate is equally distributed between males and females^7^. Uveal melanoma exhibits distinct epidemiological and pathophysiological characteristics compared with cutaneous melanoma. While ultraviolet (UV) light is a key risk factor for cutaneous forms, its role in uveal melanoma is limited because the posterior eye is shielded from UV radiation; however, iris melanomas, which develop in the anterior chamber and lack this protection, may be influenced by UV exposure^8,9^.

## Clinical background

Clinically, half of the patients with uveal melanoma are asymptomatic and the tumour is detected during routine eye exams, whereas symptomatic presentations often include visual disturbances or painless vision loss^10^. Diagnosis involves fundoscopy and imaging modalities, such as ultrasonography and optical coherence tomography, with biopsy mainly reserved for prognostic purposes. Historically, enucleation was the standard treatment for uveal melanoma, particularly for larger tumours. However, findings from the Collaborative Ocular Melanoma Study (COMS) demonstrated no survival benefit in patients treated with enucleation compared with brachytherapy^11^. Plaque brachytherapy with radioisotopes like ruthenium-106 or iodine-125 brachytherapy is now widely recognized as an effective treatment for small- to medium-sized tumours, achieving a 5-year local recurrence rate of 10%^12,13^. In addition, proton beam radiotherapy has shown consistently high local control rates and is considered a standard treatment approach^14^. Adjuvant systemic therapy is not routinely recommended in non-metastatic uveal melanoma, as this historically has not demonstrated efficacy in preventing metastases^15^. The ATOM trial is currently enrolling patients to evaluate whether adjuvant treatment with tebentafusp can prevent the development of distant metastases (ClinicalTrials.gov NCT06246149). Surveillance intensity may be stratified according to metastatic risk: low-risk patients may be considered for annual imaging, whereas high-risk patients could undergo hepatic imaging every 3–6 months for at least 10 years^15,16^. It is important to note that enhanced surveillance may detect earlier and smaller hepatic lesions, but this has not yet translated into an improved overall survival (OS)^17^.

## Molecular genetic background

Uveal melanoma exhibits a distinct genetic landscape that differentiates it from other melanoma subtypes. It is characterized by recurrent oncogenic mutations and chromosomal copy number aberrations that result in the transformation of melanocytes in the uveal tract to melanoma. The majority of patients with uveal melanoma (nearly 90%) carry a mutually exclusive mutation in either the *GNAQ* or *GNA11* gene^18^. These mutations result in the activation of several downstream signalling cascades, including the mitogen-activated protein kinase (MAPK), phosphatidylinositol 3-kinase (PI3K), protein kinase B (AKT), and yes-associated protein 1 (YAP1) pathways^19–21^. Chromosomal aberrations play a critical role in uveal melanoma tumorigenesis and progression. Monosomy of chromosome 3 is a hallmark of aggressive uveal melanoma and is strongly associated with metastatic risk. This alteration often coincides with mutations in the *BAP1* gene (encoding breast cancer gene 1-associated protein 1), located on chromosome 3. *BAP1* mutations lead to the loss of tumour suppressor functions, impacting chromatin remodelling, DNA repair, and cell cycle regulation, thereby promoting tumour progression^22^. These mutations are found in 47% of primary uveal melanomas and in 84% of those that metastasize, and there is a strong association between *BAP1* mutation and a poor prognosis^23^ (*Fig. 1*). More recently a chromosome 8q gain has been proposed as a biomarker for poor prognosis. A retrospective study among 86 patients found a disease-free interval of <24 months and a chromosome 8q surgain (>3 copies) to be the only independent predictors for recurrence-free survival (RFS) and OS after the resection of liver metastasis^24^.

## Liver metastases from uveal melanoma

Although primary uveal melanoma lesions respond favourably to initial treatment, the prognosis for patients with metastatic disease remains poor. Distant metastases occur in 25–31% of patients within 5 years of diagnosis, 34–45% within 15 years, and around 50% within 25 years^13,25^. Metastatic spread in uveal melanoma predominantly involves the liver, which is affected in approximately 90% of the patients. Other common metastatic sites include the lungs (24%), bones (16%), and soft tissues (11%)^26^. Based on these figures, approximately five to six individuals per million in Northern Europe will develop liver metastases from uveal melanoma each year. However, this estimate reflects cumulative risks among those who survive long enough for metastases to occur. Although the timing of metastatic onset varies, metastases are typically detected 3 years after the initial diagnosis of the primary tumour, with a median age of presentation of around 63 years^27–29^. After metastases occur, the median OS is approximately 1 year, with a 2-year survival rate of only 8%^30^. Given the poor prognosis associated with metastatic uveal melanoma, early identification of high-risk patients after primary treatment may facilitate personalized management strategies.

### Risk factors for developing metastasis

The risk of metastasis is influenced by tumour size, location, and genetic profile. The current AJCC staging system for uveal melanoma is based exclusively on clinical features^31,32^. Advancing AJCC stage in uveal melanoma, determined by tumour size (largest basal diameter and thickness), ciliary body involvement, and/or extraocular extension, is strongly correlated with metastatic risk; 10-year metastasis-free survival (MFS) decreases from 95% in AJCC stage I to <60% in AJCC stage III^31^. Gene expression profiling (GEP) also enables risk stratification. GEP measures the activity of a set of genes to classify the tumour into molecular subgroups. Patients are categorized as class 1A (low metastatic risk; 5-year MFS of 98%), class 1B (intermediate metastatic risk; 5-year MFS of 79%), or class 2 (high metastatic risk; 5-year MFS of 28%). Approximately 40% of patients fall into the latter group. Notably, about 15% of patients with uveal melanoma who develop metastatic disease belong to the low-risk molecular subgroup (class 1), but can be distinguished from non-metastatic class 1 patients by elevated expression of the tumour-associated antigen preferentially expressed antigen in melanoma (PRAME)^33,34^. Genotypic profiling focuses on analysing specific genetic alterations or chromosomal aberrations in tumour DNA and plays a significant role in prognostication of uveal melanoma patients. Key genetic alterations commonly assessed include monosomy 3, gain of chromosome 8q, and mutations in genes such as *BAP1*, *SF3B1*, and *EIF1AX*^15^. The Liverpool Uveal Melanoma Prognosticator Online (LUMPO) is an online tool that combines clinicopathological features with cytogenetics to calculate the risk of developing distant metastasis for individual patients within certain time frames^35^.

### Metastatic spread in uveal melanoma

Uveal melanoma spreads almost exclusively through the haematogenous route. The choroid consists of a vascular layer without a true basement membrane; the melanocytes are embedded in a loose connective tissue environment, rather than being anchored to a basement membrane. Therefore, unlike cutaneous and mucosal melanomas, which have to invade blood vessels through a basal membrane, uveal melanomas lack this barrier, which could be one of the reasons why there is a high frequency (10–88%) of circulating tumour cells (CTCs) in patients with uveal melanoma^36–40^. These cells also have a strong and not well-understood tendency to spread to the liver, which supports the ‘seed and soil’ theory^41^. An important part of this is the development of pre-metastatic niches; recent research has focused on the role of extracellular vesicles (EVs).

Tumour-derived EVs have been shown to modulate hepatic stellate cells, induce fibronectin expression in hepatocytes, and stimulate angiogenesis via endothelial cells, collectively promoting a pro-metastatic microenvironment. These vesicles carry a range of oncogenic signals, including proteins, lipids, and nucleic acids, that activate critical signalling pathways, creating favourable conditions for tumour cell colonization^42,43^. Recent work has further emphasized the role of exosome-mediated crosstalk between uveal melanoma cells and stromal components of the liver, showing that EVs selectively home to hepatic tissue and reprogram local cells to support invasion and immune evasion^44^. Also, unique microRNA (miRNA) signatures in EVs derived from the liver circulation during isolated hepatic perfusion (IHP) indicate dynamic, tissue-specific communication that may precondition the liver for metastasis^45^.

### Growth patterns of liver metastasis

When liver metastases occur, two different growth patterns have been proposed: an infiltrative pattern and a nodular pattern^46,47^. In the infiltrative pattern, cancer cells initially colonize the sinusoidal spaces, subsequently proliferating to destroy adjacent hepatocytes and form macrometastases. On radiological imaging, these lesions often appear diffusely distributed throughout the liver parenchyma. In the nodular pattern, micrometastases initially localize near portal venules, then expand and destroy adjacent hepatocytes to form avascular nodules that eventually evolve into vascularized macrometastases. On imaging, these lesions typically present as well-circumscribed, rounded nodules in distinct regions of the liver^48^. Assuming that uveal melanoma micrometastases are seeded early and frequently, with a dormant phase occurring before the development of macrometastases, it is likely that many patients who do not present with macrometastases still have micrometastases, primarily localized in the liver. A recent study investigated the presence and characteristics of micrometastases in tissue obtained at autopsy from 11 patients with uveal melanoma. Micrometastases were identified in five of five patients with concurrent macrometastases and in five of six patients without concurrent macrometastases. This suggests that micrometastases are present in nearly all patients diagnosed with primary uveal melanoma^49^.

## Systemic treatment options for liver metastases from uveal melanoma

Although several systemic treatments have been proven effective in cutaneous melanoma, the outcomes in patients with uveal melanoma have been disappointing. This may be explained by the lower tumour mutational burden in patients with uveal melanoma compared with patients with cutaneous melanoma^50^. Consequently, therapies that have revolutionized the treatment of cutaneous melanoma, such as immune checkpoint inhibitors, often show limited effectiveness in metastatic uveal melanoma or benefit only a small group of patients, especially those with extrahepatic disease.

### Chemotherapy

Several chemotherapy agents, including dacarbazine, temozolomide, cisplatin, and treosulfan, have been investigated in metastatic uveal melanoma, but their efficacy remains limited. *Table 1* shows a summary of all prospective single-arm studies and RCTs of chemotherapeutic agents. The median progression-free survival (PFS) among 238 patients included in eight studies between 2004 and 2020 ranged between 1.8 and 5.8 months, while the median OS among 258 patients included in nine studies ranged between 7.7 and 17.0 months^51–59^.

**Table 1:** Prospective single-arm studies and RCTs of systemic treatments in patients with metastatic uveal melanoma

Study	Patients	Study design	Treatment	Median PFS (months)	Median OS (months)
**Chemotherapy**					
Carvajal *et al*.^51^, 2018	32	Prospective randomized phase III trial	Dacarbazine	1.8	8.6
Corrie *et al*.^52^, 2005	5	Prospective NRCS phase I	Gemcitabine + treosulfan	5.8	12.2
Homsi *et al*.^53^, 2010	22	Prospective NRCS phase II	Docosahexaenoic acid-paclitaxel	3.0	9.8
Leyvraz *et al*.^54^, 2014	85	Prospective randomized trial EORTC multicentre	Fotemustine	4.5	13.8
Luke *et al*.^55^, 2020	15	Prospective randomized phase II trial	Temozolomide or dacarbazine	2.0	7.3
Piperno-Neumann *et al*.^56^, 2016	35	Prospective NRCS phase II	Temozolomide + bevacizumab	3.0	10.0
Schinzari *et al*.^57^, 2017	25	Prospective NRCS phase II	Cisplatin + dacarbazine + vinblastine	5.5	13.0
Schmittel *et al*.^58^, 2005	19	Prospective NRCS phase II	Gemcitabine + treosulfan + cisplatin	3.0	7.7
Terheyden *et al*.^59^, 2004	20	Prospective NRCS phase II	Gemcitabine + treosulfan	NR	17.0
**Targeted therapy**					
Carvajal *et al*.^60^, 2014	50	Prospective randomized phase III trial	Selumetinib	4.0	11.8
Carvajal *et al*.^51^, 2018, SUMIT trial	97	Prospective randomized phase III trial	Selumetinib + dacarbazine	2.8	10.1
Daud *et al*.^61^, 2017	23	A prospective randomized discontinuation trial	Cabozantinib	4.8	12.6
Hofmann *et al*.^62^, 2009	12	Prospective NRCS phase II	Imatinib	NR	5.5*
Luke *et al*.^55^, 2020	31	Prospective randomized phase II trial	Cabozantinib	2.0	6.4
Mahipal *et al*.^63^, 2012	20	Prospective NRCS pilot study	Sunitinib	4.2	8.2
Mouriax *et al*.^64^, 2016	32	Prospective trial phase II	Sorafenib	2.5*	7.8*
Piperno-Neumann *et al*.^65^, 2020	153	Prospective NRCS phase I	Sotrastaurin	3.5	NR
Piperno-Neumann *et al*.^66^, 2023	68	Prospective NRCS phase I	Darovasertib	3.6	NR
Penel *et al*.^67^, 2008	13	Prospective trial phase II	Imatinib mesylate	NR	10.8
Sacco *et al*.^68^, 2024	77	Prospective randomized phase II trial	Selumetinib	3.4	10
Shah *et al*.^69^, 2018	17	Prospective trial phase II	Ganetespib	1.7	6.7
**Immune checkpoint inhibition**					
Danielli *et al*.^70^, 2012	13	Prospective NRCS	Ipilimumab	NR	8.3
Joshua *et al*.^71^, 2015	11	Prospective trial phase II	Tremelimumab	2.9	12.8
Maio *et al*.^72^, 2013	82	Prospective NRCS	Ipilimumab	3.6	6.0
Ny *et al*.^73^, 2021	29	Prospective trial phase II	Pembrolizumab + entinostat	2.1	13.4
Pelster *et al*.^74^, 2021	33	Prospective trial phase II	Nivolumab + ipilimumab	5.5	19.1
Piulats *et al*.^75^, 2021	52	Prospective trial phase II	Nivolumab + ipilimumab	3.0	12.7
Rossi *et al*.^76^, 2019	16	Prospective NRCS	Pembrolizumab	3.8	Not reached
Rozeman *et al*.^77^, 2020	38	Prospective trial phase Ib/II	Ipilimumab 10 mg/kg + RFA Ipilimumab 3 mg/kg + RFA	3.0 3.0	14.2 9.7
Zimmer *et al*.^78^, 2015	52	Prospective NRCS phase II	Ipilimumab	2.8	6.8
**Tebentafusp**					
Carvajal *et al*.^79^, 2022	42	Prospective trial phase I/II	Tebentafusp	4.6	25.5
Carvajal *et al*.^80^ , 2022	127	Prospective trial phase II	Tebentafusp	8.7	16.8
Hassel *et al*.^81^, 2023	252	Prospective randomized phase III trial	Tebentafusp	3.3	21.6

PFS, progression-free survival; OS, overall survival; NRCS, non-randomized case series; EORTC, European Organisation for Research and Treatment of Cancer; NR, not reported; RFA, radiofrequency ablation. *Estimated from Kaplan–Meier curves. †Only abstract available; the total group included 77 patients and unclear how many were randomized to selumetinib alone.

### Targeted therapy

Targeted therapy is an approach that selectively inhibits cancer cell growth or survival, either by blocking proliferative signalling pathways or by enhancing mechanisms that induce tumour cell death^20^. Evidence from preclinical studies indicated that mitogen-activated protein kinase kinase (MEK) inhibition is effective against uveal melanoma cells *in vitro*. This finding initiated three RCTs, evaluating the MEK inhibiter selumetinib^51,60,68^. While two of the three trials demonstrated a significant increase in PFS, neither showed a benefit in OS^60,68^. Protein kinase C (PKC) inhibitors represent a promising therapeutic strategy, due to their ability to target key oncogenic signalling pathways driven by *GNAQ* and *GNA11* mutations. The PKC inhibitor LXS196 (darovasertib) has demonstrated encouraging clinical activity with manageable toxicity in a phase I multicentre study among 68 patients^66^. Another PKC inhibitor, AEB071 (sotrastaurin), showed modest clinical selective activity and was well tolerated in a phase I clinical trial, including 153 patients^65^. Prospective single-arm studies and RCTs with targeted therapies, with corresponding survival data, are summarized in *Table 1*, showing similar PFS and OS data compared with studies performed with chemotherapeutic agents. The median PFS among 269 patients included in eight studies between 2008 and 2020 ranged between 1.7 and 4.8 months, while the median OS among 295 patients included in ten studies ranged between 5.5 and 12.6 months.

### Immune checkpoint inhibition

After the successful introduction of immune checkpoint inhibition in the treatment of cutaneous melanoma, attempts have been made to extend these findings to uveal melanoma. However, the low tumour mutational burden in uveal melanoma may explain its reduced sensitivity to these treatments. The median tumour mutational burden in patients with uveal melanoma was found to be 1.3 mutations per megabase, ranking among the lowest 5% across 168 analysed cancer types^82^. A recent study comparing metastatic uveal melanoma and cutaneous melanoma found that uveal melanoma liver metastases show lower levels of immune checkpoint inhibitor response markers, highlighting the immunologically colder tumour microenvironment compared with cutaneous melanoma metastases^83^. Between 2012 and 2015, five single-arm studies evaluated the use of the cytotoxic T-lymphocyte-associated protein-4 (CTLA-4) inhibitor ipilimumab or the programmed death-ligand 1 (PD-1) inhibitors tremelimumab and pembrolizumab as monotherapy. The median PFS ranged between 2.8 and 3.8 months, while the median OS ranged between 6.0 and 12.8 months, demonstrating outcomes comparable to those observed in studies on targeted therapies and chemotherapy^70–72,76,78^. The PEMDAC trial aimed to improve the efficacy of the PD-1 inhibitor pembrolizumab by adding an epigenetic therapy using the histone deacetylase (HDAC) inhibitor entinostat^73^. This approach led to a median PFS of 2.1 months and a median OS of 13.4 months. Two single-arm trials have evaluated the combination of CTLA-4 and PD-1 inhibition (ipilimumab and nivolumab), showing a median PFS of 3.0 and 5.5 months and a median OS of 12.7 and 19.1 months respectively^74,75^ (*Table 1*). Incorporating data from four retrospective cohort studies, involving a total 174 patients, the median PFS ranged between 2.7 and 3.5 months, while the median OS varied between 15 and 18.9 months^84–87^.

### Tebentafusp

The first treatment to demonstrate an improvement in OS in a phase III randomized trial was tebentafusp, a bispecific antibody designed to enhance immune targeting by fusing an affinity-enhanced T cell receptor with an anti-CD3 effector, thereby redirecting T cells to recognize and target glycoprotein 100 (gp100)-positive melanoma cells. However, patients must have an HLA-A2*02:01 genotype to be eligible for treatment, a genotype present in approximately 50% of the Western population^81^. In the trial, participants were randomized 2 : 1 to receive either tebentafusp or an investigator-selected treatment of pembrolizumab, ipilimumab, or dacarbazine. The study included 252 patients treated with tebentafusp and 126 patients who received the investigator-selected treatment, and the results showed an improvement in OS for the tebentafusp group, with a median survival of 21.6 months, compared with 16.9 months for the control group. Although OS was significantly improved, the effects on PFS and tumour shrinkage were minimal, resulting in approximately 2–3 weeks of a PFS benefit in favour of the tebentafusp group^81^. These findings suggest that while tebentafusp may not lead to a significant reduction in tumour size, it still provides an approximately 4.5-month benefit in terms of OS. The trial also showed that circulating tumour DNA (ctDNA) may serve as a more reliable predictor of treatment efficacy than radiographic assessments, as it has been associated with improved OS, even in cases where response evaluation criteria in solid tumors indicated progressive disease or stable disease^88^. Furthermore, outcome data from two additional single-arm studies further support the efficacy of tebentafusp in patients with uveal melanoma metastases (*Table 1*)^79,80^.

## Locoregional therapies for liver metastases from uveal melanoma

As metastatic disease predominantly involves the liver, a wide range of treatment strategies that specifically target the liver have been implemented. In oligometastatic disease, these treatment strategies include metastasectomy, thermal ablation therapies, and stereotactic body radiation therapy (SBRT). However, in the majority of patients, these ablative procedures are not suitable due to a more extensive intrahepatic disease or a miliary pattern of spread in the liver. Different types of intra-arterial treatments can then be considered, including hepatic arterial infusion (HAI), transarterial chemoembolization (TACE), selective internal radiation therapy (SIRT), IHP, or percutaneous hepatic perfusion (PHP).

### Surgical resection

Surgical resection (that is hepatectomy) may offer a clinical benefit in terms of increased median OS in highly selected patients. These patients typically develop hepatic metastases after a longer interval (≥24 months) after primary uveal melanoma diagnosis and present with a limited number of lesions (absence of miliary disease and ≤4 lesions) and no extrahepatic metastases^89^.

Unlike colorectal liver metastases, where resection is pursued aggressively when complete clearance is feasible, the approach in uveal melanoma remains more cautious due to the frequent miliary spread and poor overall prognosis. Even if patients have resectable findings on imaging, approximately 30% of patients are found to have unresectable disease at the time of surgical exploration^90^. Furthermore, traditional practice has excluded patients with extrahepatic metastases from resection; however, emerging data challenge this view. Patients with only extrahepatic metastases have a longer OS than those with hepatic involvement, potentially supporting a strategy to prioritize control of hepatic disease, even in the presence of limited extrahepatic involvement^91^. Additionally, a recent review reported superior outcomes for patients undergoing surgery or ablation, though this is likely reflective of selection bias, favouring patients with more indolent disease^92^. A systematic review of data from ten cohort studies reported a median OS of 10–35 months for surgically treated patients *versus* 9–15 months for those receiving systemic chemotherapy or best supportive care. It is important to acknowledge the heterogeneity among these studies, as, in half of them, surgically treated patients also received additional locoregional therapies (for example HAI or TACE). Consequently, survival outcomes may be influenced by selection bias, with patients exhibiting less extensive and aggressive metastatic disease more likely to be eligible for surgical resection^93^. Together, these findings suggest that while liver resection remains an option for select patients, clear indications and prospective validation are lacking, and multidisciplinary evaluation is essential.

### SBRT

SBRT is a highly precise radiation technique that delivers high-dose radiation to tumours in a limited number of fractions, minimizing damage to surrounding healthy tissue. Most studies on SBRT for liver metastases include a heterogeneous patient population with various primary tumours, often without specific data on uveal melanoma. A single-centre study of 33 patients included 9 patients with melanoma (however, it was not specified whether any had uveal melanoma); a relatively high expression index for radiosensitivity in treated melanoma lesions was found^94^. Another small retrospective single-centre study, which only included uveal melanoma patients with liver metastases, compared 13 patients who received SBRT within 6 months of diagnosis (radiation therapy group) with 11 patients who did not receive SBRT within 6 months (no radiation therapy group). The median OS for patients in the radiation therapy group was 13 months compared with 39 months for patients in the no radiation therapy group (*P* = 0.02). However, the rationale for initiating SBRT in these patients was not specified, potentially introducing selection bias and impacting OS outcomes^95^.

### Thermal ablation

Thermal ablation techniques, including radiofrequency ablation (RFA), microwave ablation (MWA), and cryoablation, are minimally invasive, percutaneous, image-guided procedures that cause thermally induced coagulation necrosis of the target tissue. Both RFA and MWA cause thermally induced coagulative necrosis of the target tissue, while cryoablation, in contrast, utilizes tissue freezing to induce cellular damage^96^. Thermal ablation in uveal melanoma liver metastases is limited to patients with oligometastatic disease. The indication for thermal ablation thus overlaps with that of surgical resection, but there is increasing interest in minimally invasive interventions for oligometastatic lesions^97^. In the small number of available retrospective studies comparing ablation *versus* surgery, no differences in median OS or disease-free survival have been found between the treatment groups^96^.

### Transcatheter intra-arterial embolization

**Fig. 2:**
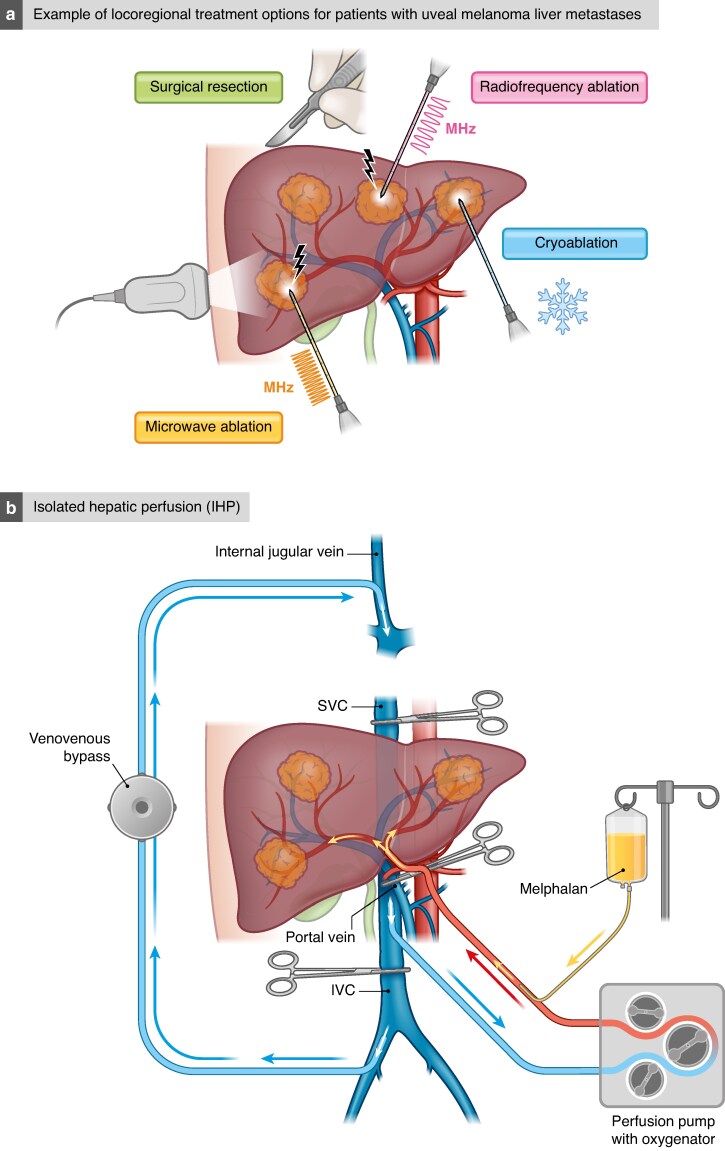

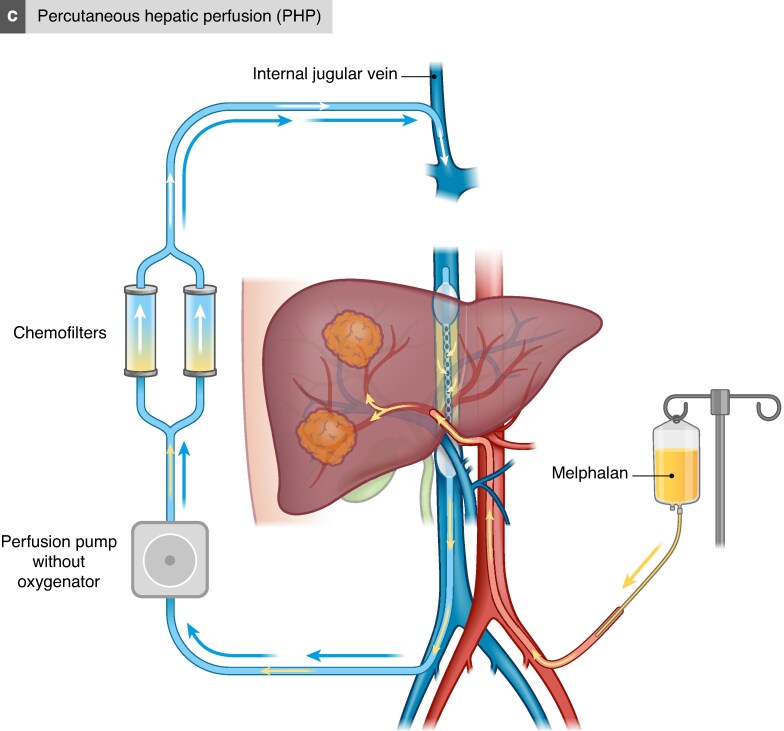
Locoregional therapeutic options for uveal melanoma liver metastases **a** Example of locoregional treatment options for patients with uveal melanoma liver metastasis. **b** Isolated hepatic perfusion (IHP). **c** Percutaneous hepatic perfusion (PHP).

With more extensive intrahepatic disease involvement, transarterial treatment strategies can be considered. These can be further divided into embolic or non-embolic approaches^15^. These therapies mainly take advantage of the fact that both primary and secondary liver tumours derive their blood supply from the hepatic artery^98^. In the case of the embolic approaches, metastases can be made ischaemic, while non-malignant liver tissue is mainly spared. During the past decades, transcatheter intra-arterial embolization procedures have become more established in the management of various conditions, especially for the treatment of primary and secondary malignancies in the liver. Though bland embolization was performed in the past, the most common treatment methods used today are TACE and SIRT. See *Fig. 2a*.

TACE involves lipiodol and/or particle embolization of the metastases, along with the delivery of local chemotherapeutic agents. In conventional TACE, chemotherapeutic agents are mixed with lipiodol, an ethiodized oil that acts as a drug-delivering carrier and as an embolic agent^96^. Over the years, drug-eluting beads (DEBs) have been developed as another variation of drug delivery (aka DEB-TACE). These DEBs release the chemical compounds in a controlled manner, reducing systemic toxicities^96^. One systematic review evaluated 17 studies with widely varying use of chemotherapeutic agents and treatment protocols in patients with uveal melanoma; a median OS ranging between 5 and 29 months was reported^93^.

SIRT uses a transcatheter approach to deliver Yttrium-90 or Holmium-166 labelled microspheres (made of either glass or resin) that are labelled with yttrium-90 or holmium-166 via the hepatic artery^96^. These microspheres are predominantly β-emitters, with a maximum soft-tissue penetration of 11 mm. The aim is to deliver high-dose radiation to the lesions, while minimizing damage to the normal liver tissue. The use of SIRT in liver-metastasized uveal melanoma was first described in a retrospective multicentre study in 2009^99^. In a prospective two-parallel arm phase II trial, treatment-naive patients and patients who progressed after immunoembolization were treated with SIRT, showing a median PFS of 8.1 and 5.2 months respectively and a median OS of 18.5 and 19.2 months respectively^100^. A small number of retrospective cohort studies have followed since then, reporting a median OS ranging between 9 and 24 months^93^.

### IHP

IHP is an open surgical procedure where the liver is isolated from the systemic circulation and perfused with a chemotherapeutic agent under hyperthermic conditions. Laparotomy is performed, allowing positioning of an inflow catheter in the proper hepatic artery and an outflow catheter in the inferior caval vein. These catheters are then connected to a heart–lung machine and the liver is perfused with a high dose of chemotherapy as a one-time treatment^101^ (*Fig. 2b*). In the phase III randomized controlled SCANDIUM trial, comparing IHP with best alternative care (BAC), the results showed an overall response rate of 40% with IHP compared with 4.5% with BAC^102^, leading to a statistically significant benefit in median PFS of 7.4 months compared with 3.3 months and a numerically, but statistically non-significant, benefit in OS of 21.7 months compared with 17.6 months^103^.

### PHP

In the early 1990s, independent groups developed a novel PHP system using extracorporeal chemofiltration. PHP is performed under general anaesthesia and permits percutaneous isolation of the liver from the systemic circulation^104,105^. The main advantages of PHP over IHP are that it is a minimally invasive method, reducing the surgical trauma, and that it can be repeated several times. A double-balloon catheter is inserted via the right common femoral vein and positioned in the inferior caval vein, with the cranial balloon inflated at the atriocaval junction and the caudal balloon above the renal veins. The catheter is then connected to an extracorporeal circulation system, consisting of a centrifugal pump and an activated carbon drug filtration unit. After placement of the chemotherapy infusion catheter in the hepatic artery, melphalan is infused and the effluent chemosaturated blood is aspirated through catheter fenestrations between the balloons of the double-balloon catheter and pumped through the filtration system to remove melphalan from the blood. The cleaned blood is then returned to the systemic circulation through a catheter in the internal jugular vein (*Fig. 2c*). The largest prospective single-arm study, including 64 PHP procedures performed between 2014 and 2017 in 35 patients, reported a median PFS of 7.6 months, with a median OS of 20.5 months^106^. The randomized controlled FOCUS trial compared PHP with the investigator’s choice of TACE, ipilimumab, pembrolizumab, or dacarbazine. The primary endpoint was PFS and the trial showed a benefit for PHP, with a median PFS of 9.1 months compared with 3.3 months (*P* < 0.004)^107^. The overall response rate was nearly tripled in favour of PHP (36.3% compared with 12.5%; *P* < 0.012) and the disease control rate was also significantly in favour of the PHP group (73.6% compared with 37.5%; *P* < 0.0002). However, it is important to note that the BAC arm was discontinued part way through the study secondary to patients’ refusal to be treated on the BAC arm, and subsequently withdrawing from the study in order to seek PHP in Europe where it was CE mark approved.

When comparing IHP with PHP, a meta-analysis, including a total of 292 patients, found that there was no difference in OS or PFS between the two treatments, but that there was a significantly lower risk of complications and 30-day mortality after PHP compared with IHP^108^.

### HAI

HAI therapy has also been explored as a treatment modality, leveraging on the preferential arterial blood supply of hepatic tumours. Compared with IHP or PHP, which achieve high hepatic drug concentrations at a single time point, HAI achieves a continuous low-dose infusion via an implantable pump, typically over weeks to months, and is commonly used in colorectal liver metastases. A previous systematic review of HAI for patients with uveal melanoma liver metastases identified eight studies, with a total of 405 patients, treated primarily with fotemustine. The reported median OS was 10–24 months^93^. In an RCT of 171 patients comparing fotemustine delivered systemically or by HAI, there was no difference in OS (14 *versus* 15 months respectively). However, HAI was associated with a longer PFS (4.5 *versus* 3.7 months respectively)^54^. The role of HAI remains less clear, but may still represent a viable liver-directed option in specialized centres or in combination with systemic treatments.

## Systemic immunotherapy with locoregional treatment

An interesting development is the combination of IHP or PHP with systemic immunotherapy. The addition of immunotherapy aims to provide a beneficiary cumulative effect and improve the clinical outcomes of the locoregional therapy, by either preventing tumour cell-mediated suppression of the immune system or releasing tumour-specific antigens, making tumour cells more vulnerable to targeted killing by the immune system. This approach was investigated in the SCANDIUM II trial, combining IHP with ipilimumab 3 mg/kg and nivolumab 1 mg/kg (IPI3/NIVO1), and the results showed that the combination was associated with severe adverse events, but with promising efficacy when the immunotherapy was given after the IHP^109^. Another trial is the ongoing CHOPIN trial, where PHP is combined with the flipped dose of ipilimumab 1 mg/kg and nivolumab 3 mg/kg (IPI1/NIVO3)^110^, and data reported from the first seven patients showed very promising efficacy, with an overall response rate of 85.7% and a disease control rate of 100%. Currently, the randomized SCANDIUM III trial compares PHP with adjuvant IPI1/NIVO3 with systemic IPI3/NIVO1 only (ClinicalTrials.gov NCT05648110) (*Table 2*). To take advantage of the possible synergy between radiation and immunotherapy, Minor *et al*.^111^ treated patients with two treatments of SIRT followed by four doses of combinational immunotherapy with ipilimumab/nivolumab and then maintenance nivolumab. Initial results of 13 patients showed a median PFS of 27 weeks and a median OS of 48 weeks, but with a high rate of grade 3–4 hepatic toxicities, which resulted in a protocol change to a reduced dose of postoperative ipilimumab from 3 mg/kg to 1 mg/kg^111^. Another interesting development is, for example, the PERIO-01 trial, a phase I/II study investigating the intrahepatic delivery of a toll-like receptor-9 agonist (nelitolimod) via HAI, in combination with systemic checkpoint inhibition, aiming to enhance local immune activation and overcome liver-specific immunosuppression (ClinicalTrials.gov NCT04935229).

**Table 2:** RCTs of locoregional therapies of the liver and those combined with systemic immunotherapy

Study	Patients	Study design	Treatment	Median PFS (months)	Median OS (months)
**Locoregional therapies**					
Olofsson Bagge *et al*.^103^, 2024, SCANDIUM trial	93	Prospective randomized phase III trial	Isolated hepatic perfusion with melphalan *versus* best alternative care	7.4 *versus* 3.3 (*P* < 0.0001)	21.7 *versus* 17.6 (NS; *P* value NR)
Peuker *et al*.^112^, 2022, SirTac trial	40	Prospective randomized phase II trial	Selective internal radiation therapy *versus* transarterial chemoembolization using cisplatin	4.9 *versus* 2.2 (*P* = 0.037)	NR
Valsecci *et al*.^113^, 2015	52	Prospective randomized phase II trial	Immunoembolization using GM-CSF *versus* bland embolization	10.4 *versus* 7.1 (NS)	21.5 *versus* 17.2 (*P* = 0.047)
Zager *et al*.^107^, 2024, FOCUS trial	123	Prospective randomized phase III trial	Percutaneous hepatic perfusion with melphalan *versus* best alternative care	9.1 *versus* 3.3 (*P* > 0.004)	20.5 *versus* 14.1 (NS; *P* value NR)
**Locoregional therapies combined with systemic immunotherapy**					
Olofsson Bagge *et al*.^109^, 2024, SCANDIUM II trial	18	Prospective randomized phase Ib trial	Isolated hepatic perfusion followed by four cycles of IPI3/NIVO1 (postoperative arm) *versus* IPI3/NIVO1 for one cycle, then isolated hepatic perfusion followed by three cycles of IPI3/NIVO1 (preoperative/postoperative arm)	11.8 *versus* 6.0 (NS; *P* value NR)	NR
Tong *et al*.^110^, 2023, CHOPIN trial	83	Combined phase Ib (7 patients)/randomized phase II (76 patients) trial	Percutaneous hepatic perfusion with melphalan + IPI/NIVO, dose-escalation design (phase Ib) Percutaneous hepatic perfusion with melphalan *versus* percutaneous hepatic perfusion with melphalan + IPI1/NIVO3 (phase II)	29.1 (phase Ib) Ongoing (phase II)	NR (phase Ib) Ongoing (phase II)

PFS, progression-free survival; OS, overall survival; NS, non-significant; NR, not reported; GM-CSF, granulocyte–macrophage colony-stimulating factor; IPI1, ipilimumab 1 mg/kg; IPI3, ipilimumab 3 mg/kg; NIVO1, nivolumab 1 mg/kg; NIVO3, nivolumab 3 mg/kg.

### HAI with autologous tumour-infiltrating lymphocytes (TILs)

Treatment with autologous TILs is a novel treatment. TILs refer to T cells and/or B cells isolated directly from a tumour, either obtained by surgical resection or acquired by biopsy. The TILs are expanded ex vivo and, once successfully grown to sufficient numbers, the TILs are given back to the same patient after a course of non-myeloablative, lymphodepleting chemotherapy. Most commonly, the TIL infusion is given intravenously, but, in the phase I trial HAI-TILs (ClinicalTrials.gov NCT04812470), a minimally invasive catheter-guided HAI of the lymphocytes was performed instead. This approach could theoretically also be combined with PHP, where the PHP could replace total lymphodepleting chemotherapy andinstead mostly focusing on depleting lymphocytes in the liver.

**Fig. 3:**
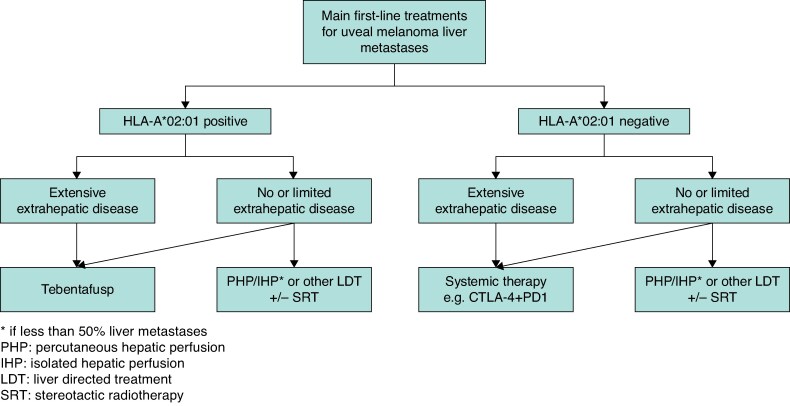
Proposed treatment algorithm for patients with uveal melanoma metastases Besides the systemic treatments listed in the algorithm, eligible patients should be considered for inclusion in clinical trials investigating novel therapies. *Selection of locoregional treatment option is based on tumour size and anatomical location, and the treatment modalities available at the institution. CTLA-4, cytotoxic T-lymphocyte associated protein-4; PD-1, programmed cell death protein 1.

While multidisciplinary discussion remains essential for all patients with uveal melanoma metastasis, taking individual patient corrections into account, the authors propose a treatment algorithm that may support clinical decision-making (*Fig. 3*).

## Emerging therapies and future directions

In addition to established and investigational systemic and locoregional therapies, several novel modalities are currently under exploration. Histotripsy, a non-invasive, non-thermal ablation technique utilizing focused ultrasonography to mechanically disrupt liver tissue through acoustic cavitation, has recently shown early promise in initial clinical trials^114^. Irreversible electroporation uses high-voltage electrical pulses to induce apoptosis, while preserving the surrounding vasculature and connective tissue, and is therefore a promising modality for the ablation of tumours near bile ducts and blood vessels, and early clinical studies suggest its feasibility and safety in the treatment of hepatic malignancies^115^. Additionally, oncolytic virotherapy, involving intratumoral injection of genetically modified viruses, such as herpes simplex virus (HSV) or adenoviruses, aims to lyse tumour cells and stimulate systemic immune responses. One such agent, RP2, an enhanced-potency oncolytic HSV-1-expressing human granulocyte-macrophage colony-stimulating factor, a Gibbon Ape Leukemia Virus glycoprotein (GALV-GP-R−), and an anti-CTLA-4 antibody-like molecule, has demonstrated a 29.4% overall response rate in a phase I trial, including 17 patients with advanced uveal melanoma, both as monotherapy and in combination with nivolumab^116^. These novel strategies offer promising avenues for future therapeutic development and may complement existing liver-directed and systemic treatments.

## Biomarkers for response

Liquid biopsies are a non-invasive diagnostic tool that can detect tumour-derived material in the circulating blood and can therefore be of value in the diagnosis, prognosis, and monitoring of cancer. In patients with uveal melanoma, circulating miRNA, CTCs, and ctDNA have been explored as potential valuable tools^117^. Serum miRNA can be of value in the diagnostic phase, differentiating a benign melanocytic lesion from primary uveal melanoma^118,119^. After the diagnosis of uveal melanoma, CTCs may serve as a prognostic test to establish the risk of metastasis, while, after treatment of the primary tumour, plasma ctDNA could serve as an early detector for uveal melanoma metastasis or to evaluate the treatment response. In the previously discussed tebentafusp phase III trial of Hassel *et al*.^81^, the radiological response underestimated the OS benefit of tebentafusp, but an early reduction in ctDNA levels at 9 weeks was strongly associated with OS.

## Conclusion

Uveal melanoma is a rare malignancy that differs from cutaneous melanoma in terms of genetic profile, metastatic behaviour, and response to treatment. Despite advances in local tumour control, the OS of patients with distant metastasis remains poor, underscoring the urgent need for effective systemic therapies. The first treatment to demonstrate an improvement in OS is tebentafusp, a bispecific antibody designed to enhance immune targeting. The liver remains the predominant site of metastasis and various locoregional treatment modalities have been explored; IHP and PHP have shown high response rates and clear benefits concerning PFS.

A future research direction is to optimize the combination of these liver-directed treatments with modern immunotherapies to further enhance efficacy. There is also a great unmet need for more individualized treatment strategies, which in the near future will be based on genetic and immunological profiling, which will also be essential for improving patient outcomes.

## Data Availability

No new data were generated or analysed in this study.

## References

[1] Krantz BA, Dave N, Komatsubara KM, Marr BP, Carvajal RD. Uveal melanoma: epidemiology, etiology, and treatment of primary disease. Clin Ophthalmol 2017;11:279–289 28203054 10.2147/OPTH.S89591 PMC5298817

[2] Bergman L, Seregard S, Nilsson B, Ringborg U, Lundell G, Ragnarsson-Olding B. Incidence of uveal melanoma in Sweden from 1960 to 1998. Invest Ophthalmol Vis Sci 2002;43:2579–2583 12147588

[3] McLaughlin CC, Wu XC, Jemal A, Martin HJ, Roche LM, Chen VW. Incidence of noncutaneous melanomas in the U.S. Cancer 2005;103:1000–1007 15651058 10.1002/cncr.20866

[4] Singh AD, Turell ME, Topham AK. Uveal melanoma: trends in incidence, treatment, and survival. Ophthalmology 2011;118:1881–1885 21704381 10.1016/j.ophtha.2011.01.040

[5] Gill V, Herrspiegel C, Sabazade S, Fili M, Bergman L, Damato B et al Trends in uveal melanoma presentation and survival during five decades: a nationwide survey of 3898 Swedish patients. Front Med (Lausanne) 2022;9:926034 35721086 10.3389/fmed.2022.926034 PMC9200980

[6] Nissen K, Kiilgaard JF, Fili M, Seregard S, Navaratnam J, Krohn J et al Increasing incidence of posterior uveal melanoma in Scandinavia 1960–2022: a tri-national study. Am J Ophthalmol 2025;274:131–141 40054546 10.1016/j.ajo.2025.03.002

[7] Hou X, Rokohl AC, Li X, Guo Y, Ju X, Fan W et al Global incidence and prevalence in uveal melanoma. Adv Ophthalmol Pract Res 2024;4:226–232 39726825 10.1016/j.aopr.2024.10.001 PMC11670701

[8] Karlsson J, Nilsson LM, Mitra S, Alsén S, Shelke GV, Sah VR et al Molecular profiling of driver events in metastatic uveal melanoma. Nat Commun 2020;11:1894 32313009 10.1038/s41467-020-15606-0 PMC7171146

[9] Logan P, Bernabeu M, Ferreira A, Burnier MN Jr. Evidence for the role of blue light in the development of uveal melanoma. J Ophthalmol 2015;2015:386986 26075084 10.1155/2015/386986 PMC4449937

[10] Damato EM, Damato BE. Detection and time to treatment of uveal melanoma in the United Kingdom: an evaluation of 2,384 patients. Ophthalmology 2012;119:1582–1589 22503229 10.1016/j.ophtha.2012.01.048

[11] Collaborative Ocular Melanoma Study Group . The COMS randomized trial of iodine 125 brachytherapy for choroidal melanoma: V. Twelve-year mortality rates and prognostic factors: COMS Report No. 28. Arch Ophthalmol 2006;124:1684–1693 17159027 10.1001/archopht.124.12.1684

[12] Jampol LM, Moy CS, Murray TG, Reynolds SM, Albert DM, Schachat AP et al The COMS randomized trial of iodine 125 brachytherapy for choroidal melanoma: IV. Local treatment failure and enucleation in the first 5 years after brachytherapy. COMS report no. 19. Ophthalmology 2002;109:2197–2403 12466159 10.1016/s0161-6420(02)01277-0

[13] Diener-West M, Reynolds SM, Agugliaro DJ, Caldwell R, Cumming K, Earle JD et al Screening for metastasis from choroidal melanoma: the Collaborative Ocular Melanoma Study Group Report 23. J Clin Oncol 2004;22:2438–2444 15197206 10.1200/JCO.2004.08.194

[14] Hrbacek J, Mishra KK, Kacperek A, Dendale R, Nauraye C, Auger M et al Practice patterns analysis of ocular proton therapy centers: the international OPTIC survey. Int J Radiat Oncol Biol Phys 2016;95:336–343 27084651 10.1016/j.ijrobp.2016.01.040

[15] Carvajal RD, Sacco JJ, Jager MJ, Eschelman DJ, Olofsson Bagge R, Harbour JW et al Advances in the clinical management of uveal melanoma. Nat Rev Clin Oncol 2023;20:99–115 36600005 10.1038/s41571-022-00714-1

[16] Hagström A, Witzenhausen H, Stålhammar G. Tailoring surveillance imaging in uveal melanoma based on individual metastatic risk. Canadian journal of ophthalmology. Journal canadien d'ophtalmologie 2025;60:e240 10.1016/j.jcjo.2024.07.014 39151896

[17] Delaney A, Yeşiltaş YS, Zabor EC, Singh AD. Surveillance for metastasis in low-risk uveal melanoma patients: the need for optimization. Ophthalmology 2025; DOI: 10.1016/j.ophtha.2025.03.019 [Epub ahead of print] 40118154

[18] Van Raamsdonk CD, Griewank KG, Crosby MB, Garrido MC, Vemula S, Wiesner T et al Mutations in *GNA11* in uveal melanoma. N Engl J Med 2010;363:2191–2199 21083380 10.1056/NEJMoa1000584 PMC3107972

[19] Chen X, Wu Q, Depeille P, Chen P, Thornton S, Kalirai H et al RasGRP3 mediates MAPK pathway activation in GNAQ mutant uveal melanoma. Cancer Cell 2017;31:685–696.e6 28486107 10.1016/j.ccell.2017.04.002 PMC5499527

[20] Ambrosini G, Musi E, Ho AL, de Stanchina E, Schwartz GK. Inhibition of mutant GNAQ signaling in uveal melanoma induces AMPK-dependent autophagic cell death. Mol Cancer Ther 2013;12:768–776 23443802 10.1158/1535-7163.MCT-12-1020

[21] Feng X, Degese MS, Iglesias-Bartolome R, Vaque JP, Molinolo AA, Rodrigues M et al Hippo-independent activation of YAP by the *GNAQ* uveal melanoma oncogene through a trio-regulated rho GTPase signaling circuitry. Cancer Cell 2014;25:831–845 24882515 10.1016/j.ccr.2014.04.016 PMC4074519

[22] Robertson AG, Shih J, Yau C, Gibb EA, Oba J, Mungall KL et al Integrative analysis identifies four molecular and clinical subsets in uveal melanoma. Cancer Cell 2018;33:151 10.1016/j.ccell.2017.12.013 29316429

[23] Harbour JW, Onken MD, Roberson ED, Duan S, Cao L, Worley LA et al Frequent mutation of *BAP1* in metastasizing uveal melanomas. Science 2010;330:1410–1413 21051595 10.1126/science.1194472 PMC3087380

[24] Mariani P, Pierron G, Ait Rais K, Bouhadiba T, Rodrigues M, Malaise D et al A clinico-genetic score incorporating disease-free intervals and chromosome 8q copy numbers: a novel prognostic marker for recurrence and survival following liver resection in patients with liver metastases of uveal melanoma. Cancers (Basel) 2024;16:3407 39410027 10.3390/cancers16193407 PMC11475758

[25] Kujala E, Mäkitie T, Kivelä T. Very long-term prognosis of patients with malignant uveal melanoma. Invest Ophthalmol Vis Sci 2003;44:4651–4659 14578381 10.1167/iovs.03-0538

[26] Collaborative Ocular Melanoma Study Group . Assessment of metastatic disease status at death in 435 patients with large choroidal melanoma in the Collaborative Ocular Melanoma Study (COMS): COMS Report No. 15. Arch Ophthalmol 2001;119:670–676 11346394 10.1001/archopht.119.5.670

[27] Valpione S, Aliberti C, Pigozzo J, Midena E, Parrozzani R, Stragliotto S et al 1140P—Metastatic uveal melanoma: a 22 years single center experience. Ann Oncol 2012; 23(Suppl 9): ix371

[28] Marshall E, Romaniuk C, Ghaneh P, Wong H, McKay M, Chopra M et al MRI in the detection of hepatic metastases from high-risk uveal melanoma: a prospective study in 188 patients. Br J Ophthalmol 2013;97:159–163 23159448 10.1136/bjophthalmol-2012-302323

[29] Xu LT, Funchain PF, Bena JF, Li M, Tarhini A, Berber E et al Uveal melanoma metastatic to the liver: treatment trends and outcomes. Ocul Oncol Pathol 2019;5:323–332 31559243 10.1159/000495113 PMC6751472

[30] Lane AM, Kim IK, Gragoudas ES. Survival rates in patients after treatment for metastasis from uveal melanoma. JAMA Ophthalmol 2018;136:981–986 29955797 10.1001/jamaophthalmol.2018.2466 PMC6142974

[31] AJCC Ophthalmic Oncology Task Force . International validation of the American Joint Committee on Cancer’s 7th edition classification of uveal melanoma. JAMA Ophthalmol 2015;133:376–383 25555246 10.1001/jamaophthalmol.2014.5395

[32] Shields CL, Di Nicola M, Bekerman VP, Kaliki S, Alarcon C, Fulco E et al Iris melanoma outcomes based on the American Joint Committee on Cancer classification (eighth edition) in 432 patients. Ophthalmology 2018;125:913–923 29342436 10.1016/j.ophtha.2017.11.040

[33] Onken MD, Worley LA, Ehlers JP, Harbour JW. Gene expression profiling in uveal melanoma reveals two molecular classes and predicts metastatic death. Cancer Res 2004;64:7205–7209 15492234 10.1158/0008-5472.CAN-04-1750 PMC5407684

[34] Onken MD, Worley LA, Char DH, Augsburger JJ, Correa ZM, Nudleman E et al Collaborative Ocular Oncology Group Report Number 1: prospective validation of a multi-gene prognostic assay in uveal melanoma. Ophthalmology 2012;119:1596–1603 22521086 10.1016/j.ophtha.2012.02.017 PMC3404209

[35] Cunha Rola A, Taktak A, Eleuteri A, Kalirai H, Heimann H, Hussain R et al Multicenter external validation of the Liverpool Uveal Melanoma Prognosticator Online: an OOG collaborative study. Cancers (Basel) 2020;12:477 32085617 10.3390/cancers12020477 PMC7072188

[36] Ulmer A, Beutel J, Süsskind D, Hilgers RD, Ziemssen F, Lüke M et al Visualization of circulating melanoma cells in peripheral blood of patients with primary uveal melanoma. Clin Cancer Res 2008;14:4469–4474 18628461 10.1158/1078-0432.CCR-08-0012

[37] Anand K, Roszik J, Gombos D, Upshaw J, Sarli V, Meas S et al Pilot study of circulating tumor cells in early-stage and metastatic uveal melanoma. Cancers (Basel) 2019;11:856 31226786 10.3390/cancers11060856 PMC6628316

[38] Callejo SA, Antecka E, Blanco PL, Edelstein C, Burnier MN Jr. Identification of circulating malignant cells and its correlation with prognostic factors and treatment in uveal melanoma. A prospective longitudinal study. Eye (Lond) 2007;21:752–759 16575415 10.1038/sj.eye.6702322

[39] Bidard FC, Madic J, Mariani P, Piperno-Neumann S, Rampanou A, Servois V et al Detection rate and prognostic value of circulating tumor cells and circulating tumor DNA in metastatic uveal melanoma. Int J Cancer 2014;134:1207–1213 23934701 10.1002/ijc.28436

[40] Suesskind D, Ulmer A, Schiebel U, Fierlbeck G, Spitzer B, Spitzer MS et al Circulating melanoma cells in peripheral blood of patients with uveal melanoma before and after different therapies and association with prognostic parameters: a pilot study. Acta Ophthalmol 2011;89:17–24 21272286 10.1111/j.1755-3768.2009.01617.x

[41] Langley RR, Fidler IJ. The seed and soil hypothesis revisited—the role of tumor-stroma interactions in metastasis to different organs. Int J Cancer 2011;128:2527–2535 21365651 10.1002/ijc.26031 PMC3075088

[42] Ambrosini G, Rai AJ, Carvajal RD, Schwartz GK. Uveal melanoma exosomes induce a prometastatic microenvironment through macrophage migration inhibitory factor. Mol Cancer Res 2022;20:661–669 34992145 10.1158/1541-7786.MCR-21-0526

[43] Urzì O, Olofsson Bagge R, Crescitelli R. Extracellular vesicles in uveal melanoma—biological roles and diagnostic value. Cancer Lett 2025;615:217531 39914771 10.1016/j.canlet.2025.217531

[44] Ramos R, Vinyals A, Campos-Martin R, Cabré E, Bech JJ, Vaquero J et al New insights into the exosome-induced migration of uveal melanoma cells and the pre-metastatic niche formation in the liver. Cancers (Basel) 2024;16:2977 39272836 10.3390/cancers16172977 PMC11394004

[45] Eldh M, Olofsson Bagge R, Lasser C, Svanvik J, Sjöstrand M, Mattsson J et al MicroRNA in exosomes isolated directly from the liver circulation in patients with metastatic uveal melanoma. BMC Cancer 2014;14:962 25510783 10.1186/1471-2407-14-962 PMC4320618

[46] Grossniklaus HE . Progression of ocular melanoma metastasis to the liver: the 2012 Zimmerman lecture. JAMA Ophthalmol 2013;131:462–469 23392528 10.1001/jamaophthalmol.2013.2547 PMC4472306

[47] Grossniklaus HE, Zhang Q, You S, McCarthy C, Heegaard S, Coupland SE. Metastatic ocular melanoma to the liver exhibits infiltrative and nodular growth patterns. Hum Pathol 2016;57:165–175 27476775 10.1016/j.humpath.2016.07.012 PMC5547398

[48] Yavuzyigitoglu S, Tang MCY, Jansen M, Geul KW, Dwarkasing RS, Vaarwater J et al Radiological patterns of uveal melanoma liver metastases in correlation to genetic status. Cancers (Basel) 2021;13:5316 34771480 10.3390/cancers13215316 PMC8582397

[49] Gill VT, Norrman E, Sabazade S, Karim A, Lardner E, Stålhammar G. Multiorgan involvement of dormant uveal melanoma micrometastases in postmortem tissue from patients without coexisting macrometastases. Am J Clin Pathol 2023;160:164–174 37052618 10.1093/ajcp/aqad029 PMC10392366

[50] Yarchoan M, Hopkins A, Jaffee EM. Tumor mutational burden and response rate to PD-1 inhibition. N Engl J Med 2017;377:2500–2501 29262275 10.1056/NEJMc1713444 PMC6549688

[51] Carvajal RD, Piperno-Neumann S, Kapiteijn E, Chapman PB, Frank S, Joshua AM et al Selumetinib in combination with dacarbazine in patients with metastatic uveal melanoma: a phase III, multicenter, randomized trial (SUMIT). J Clin Oncol 2018;36:1232–1239 29528792 10.1200/JCO.2017.74.1090

[52] Corrie PG, Shaw J, Spanswick VJ, Sehmbi R, Jonson A, Mayer A et al Phase I trial combining gemcitabine and treosulfan in advanced cutaneous and uveal melanoma patients. Br J Cancer 2005;92:1997–2003 15886706 10.1038/sj.bjc.6602586 PMC2361787

[53] Homsi J, Bedikian AY, Papadopoulos NE, Kim KB, Hwu WJ, Mahoney SL et al Phase 2 open-label study of weekly docosahexaenoic acid-paclitaxel in patients with metastatic uveal melanoma. Melanoma Res 2010;20:507–510 20881508 10.1097/CMR.0b013e3283403ce9

[54] Leyvraz S, Piperno-Neumann S, Suciu S, Baurain JF, Zdzienicki M, Testori A et al Hepatic intra-arterial versus intravenous fotemustine in patients with liver metastases from uveal melanoma (EORTC 18021): a multicentric randomized trial. Ann Oncol 2014;25:742–746 24510314 10.1093/annonc/mdt585 PMC4433517

[55] Luke JJ, Olson DJ, Allred JB, Strand CA, Bao R, Zha Y et al Randomized phase II trial and tumor mutational spectrum analysis from cabozantinib versus chemotherapy in metastatic uveal melanoma (alliance A091201). Clin Cancer Res 2020;26:804–811 31558480 10.1158/1078-0432.CCR-19-1223 PMC7055933

[56] Piperno-Neumann S, Diallo A, Etienne-Grimaldi MC, Bidard FC, Rodrigues M, Plancher C et al Phase II trial of bevacizumab in combination with temozolomide as first-line treatment in patients with metastatic uveal melanoma. Oncologist 2016;21:281–282 26911405 10.1634/theoncologist.2015-0501 PMC4786360

[57] Schinzari G, Rossi E, Cassano A, Dadduzio V, Quirino M, Pagliara M et al Cisplatin, dacarbazine and vinblastine as first line chemotherapy for liver metastatic uveal melanoma in the era of immunotherapy: a single institution phase II study. Melanoma Res 2017;27:591–595 29076951 10.1097/CMR.0000000000000401

[58] Schmittel A, Scheulen ME, Bechrakis NE, Strumberg D, Baumgart J, Bornfeld N et al Phase II trial of cisplatin, gemcitabine and treosulfan in patients with metastatic uveal melanoma. Melanoma Res 2005;15:205–207 15917703 10.1097/00008390-200506000-00010

[59] Terheyden P, Bröcker EB, Becker JC. Clinical evaluation of in vitro chemosensitivity testing: the example of uveal melanoma. J Cancer Res Clin Oncol 2004;130:395–399 15160290 10.1007/s00432-004-0569-4 PMC12161860

[60] Carvajal RD, Sosman JA, Quevedo JF, Milhem MM, Joshua AM, Kudchadkar RR et al Effect of selumetinib vs chemotherapy on progression-free survival in uveal melanoma: a randomized clinical trial. JAMA 2014;311:2397–2405 24938562 10.1001/jama.2014.6096 PMC4249701

[61] Daud A, Kluger HM, Kurzrock R, Schimmoller F, Weitzman AL, Samuel TA et al Phase II randomised discontinuation trial of the MET/VEGF receptor inhibitor cabozantinib in metastatic melanoma. Br J Cancer 2017;116:432–440 28103611 10.1038/bjc.2016.419 PMC5318966

[62] Hofmann UB, Kauczok-Vetter CS, Houben R, Becker JC. Overexpression of the KIT/SCF in uveal melanoma does not translate into clinical efficacy of imatinib mesylate. Clin Cancer Res 2009;15:324–329 19118061 10.1158/1078-0432.CCR-08-2243

[63] Mahipal A, Tijani L, Chan K, Laudadio M, Mastrangelo MJ, Sato T. A pilot study of sunitinib malate in patients with metastatic uveal melanoma. Melanoma Res 2012;22:440–446 23114504 10.1097/CMR.0b013e328358b373

[64] Mouriaux F, Servois V, Parienti JJ, Lesimple T, Thyss A, Dutriaux C et al Sorafenib in metastatic uveal melanoma: efficacy, toxicity and health-related quality of life in a multicentre phase II study. Br J Cancer 2016;115:20–24 27253171 10.1038/bjc.2016.119 PMC4931363

[65] Piperno-Neumann S, Larkin J, Carvajal RD, Luke JJ, Schwartz GK, Hodi FS et al Genomic profiling of metastatic uveal melanoma and clinical results of a phase I study of the protein kinase C inhibitor AEB071. Mol Cancer Ther 2020;19:1031–1039 32029634 10.1158/1535-7163.MCT-19-0098

[66] Piperno-Neumann S, Carlino MS, Boni V, Loirat D, Speetjens FM, Park JJ et al A phase I trial of LXS196, a protein kinase C (PKC) inhibitor, for metastatic uveal melanoma. Br J Cancer 2023;128:1040–1051 36624219 10.1038/s41416-022-02133-6 PMC10006169

[67] Penel N, Delcambre C, Durando X, Clisant S, Hebbar M, Negrier S et al O-Mel-Inib: a Cancéro-pôle Nord-Ouest multicenter phase II trial of high-dose imatinib mesylate in metastatic uveal melanoma. Invest New Drugs 2008;26:561–565 18551246 10.1007/s10637-008-9143-2

[68] Sacco JJ, Jackson R, Corrie P, Danson S, Evans TRJ, Ochsenreither S et al A 3 arm randomised phase II study of the MEK inhibitor selumetinib alone or in combination with paclitaxel (PT) in metastatic uveal melanoma (UM). Ann Oncol 2024;202:114009 10.1016/j.ejca.2024.114009 38547774

[69] Shah S, Luke JJ, Jacene HA, Chen T, Giobbie-Hurder A, Ibrahim N et al Results from phase II trial of HSP90 inhibitor, STA-9090 (ganetespib), in metastatic uveal melanoma. Melanoma Res 2018;28:605–610 30211813 10.1097/CMR.0000000000000509

[70] Danielli R, Ridolfi R, Chiarion-Sileni V, Queirolo P, Testori A, Plummer R et al Ipilimumab in pretreated patients with metastatic uveal melanoma: safety and clinical efficacy. Cancer Immunol Immunother 2012;61:41–48 21833591 10.1007/s00262-011-1089-0 PMC11028946

[71] Joshua AM, Monzon JG, Mihalcioiu C, Hogg D, Smylie M, Cheng T. A phase 2 study of tremelimumab in patients with advanced uveal melanoma. Melanoma Res 2015;25:342–347 26050146 10.1097/CMR.0000000000000175

[72] Maio M, Danielli R, Chiarion-Sileni V, Pigozzo J, Parmiani G, Ridolfi R et al Efficacy and safety of ipilimumab in patients with pre-treated, uveal melanoma. Ann Oncol 2013;24:2911–2915 24067719 10.1093/annonc/mdt376

[73] Ny L, Jespersen H, Karlsson J, Alsén S, Filges S, All-Eriksson C et al The PEMDAC phase 2 study of pembrolizumab and entinostat in patients with metastatic uveal melanoma. Nat Commun 2021;12:5155 34453044 10.1038/s41467-021-25332-w PMC8397717

[74] Pelster MS, Gruschkus SK, Bassett R, Gombos DS, Shephard M, Posada L et al Nivolumab and ipilimumab in metastatic uveal melanoma: results from a single-arm phase II study. J Clin Oncol 2021;39:599–607 33125309 10.1200/JCO.20.00605 PMC8257877

[75] Piulats JM, Espinosa E, de la Cruz Merino L, Varela M, Alonso Carrión L, Martín-Algarra S et al Nivolumab plus ipilimumab for treatment-naive metastatic uveal melanoma: an open-label, multicenter, phase II trial by the Spanish Multidisciplinary Melanoma Group (GEM-1402). J Clin Oncol 2021;39:586–598 33417511 10.1200/JCO.20.00550

[76] Rossi E, Pagliara MM, Orteschi D, Dosa T, Sammarco MG, Caputo CG et al Pembrolizumab as first-line treatment for metastatic uveal melanoma. Cancer Immunol Immunother 2019;68:1179–1185 31175402 10.1007/s00262-019-02352-6 PMC6584707

[77] Rozeman EA, Prevoo W, Meier MAJ, Sikorska K, Van TM, van de Wiel BA et al Phase Ib/II trial testing combined radiofrequency ablation and ipilimumab in uveal melanoma (SECIRA-UM). Melanoma Res 2020;30:252–260 31895753 10.1097/CMR.0000000000000653

[78] Zimmer L, Vaubel J, Mohr P, Hauschild A, Utikal J, Simon J et al Phase II DeCOG-study of ipilimumab in pretreated and treatment-naive patients with metastatic uveal melanoma. PLoS One 2015;10:e0118564 25761109 10.1371/journal.pone.0118564 PMC4356548

[79] Carvajal RD, Nathan P, Sacco JJ, Orloff M, Hernandez-Aya LF, Yang J et al Phase I study of safety, tolerability, and efficacy of tebentafusp using a step-up dosing regimen and expansion in patients with metastatic uveal melanoma. J Clin Oncol 2022;40:1939–1948 35254876 10.1200/JCO.21.01805 PMC9177239

[80] Carvajal RD, Butler MO, Shoushtari AN, Hassel JC, Ikeguchi A, Hernandez-Aya L et al Clinical and molecular response to tebentafusp in previously treated patients with metastatic uveal melanoma: a phase 2 trial. Nat Med 2022;28:2364–2373 36229663 10.1038/s41591-022-02015-7 PMC9671803

[81] Hassel JC, Piperno-Neumann S, Rutkowski P, Baurain JF, Schlaak M, Butler MO et al Three-year overall survival with tebentafusp in metastatic uveal melanoma. N Engl J Med 2023;389:2256–2266 37870955 10.1056/NEJMoa2304753 PMC11188986

[82] Chalmers ZR, Connelly CF, Fabrizio D, Gay L, Ali SM, Ennis R et al Analysis of 100,000 human cancer genomes reveals the landscape of tumor mutational burden. Genome Med 2017;9:34 28420421 10.1186/s13073-017-0424-2 PMC5395719

[83] Shao YF, Baca Y, Hinton A, Xiu J, VanderWalde A, Hadfield M et al Immune profiling of uveal melanoma liver metastases and response to checkpoint inhibitors. J Immunother 2025;48:189–195 40231356 10.1097/CJI.0000000000000558 PMC12052074

[84] Najjar YG, Navrazhina K, Ding F, Bhatia R, Tsai K, Abbate K et al Ipilimumab plus nivolumab for patients with metastatic uveal melanoma: a multicenter, retrospective study. J Immunother Cancer 2020;8:e000331 32581057 10.1136/jitc-2019-000331 PMC7319717

[85] Heppt MV, Amaral T, Kähler KC, Heinzerling L, Hassel JC, Meissner M et al Combined immune checkpoint blockade for metastatic uveal melanoma: a retrospective, multi-center study. J Immunother Cancer 2019;7:299 31722735 10.1186/s40425-019-0800-0 PMC6854774

[86] Bol KF, Ellebaek E, Hoejberg L, Bagger MM, Larsen MS, Klausen TW et al Real-world impact of immune checkpoint inhibitors in metastatic uveal melanoma. Cancers (Basel) 2019;11:1489 31623302 10.3390/cancers11101489 PMC6826482

[87] Kirchberger MC, Moreira A, Erdmann M, Schuler G, Heinzerling L. Real world experience in low-dose ipilimumab in combination with PD-1 blockade in advanced melanoma patients. Oncotarget 2018;9:28903–28909 29988983 10.18632/oncotarget.25627 PMC6034742

[88] Sullivan R, Collins L, Rodrigues M, Nathan P, Hassel JC, Dummer R et al Abstract 1035: Early ctDNA reduction is associated with better overall survival in the Ph 3 trial of tebentafusp in previously untreated metastatic uveal melanoma. Cancer Res 2023; 83(Suppl): 1035

[89] Mariani P, Piperno-Neumann S, Servois V, Berry MG, Dorval T, Plancher C et al Surgical management of liver metastases from uveal melanoma: 16 years’ experience at the Institut Curie. Eur J Surg Oncol 2009;35:1192–1197 19329272 10.1016/j.ejso.2009.02.016

[90] Maspero M, Pezzoli I, Di Guardo L, Angi M, Lo Dico S, Sposito C et al Intention-to-treat analysis of hepatic resection for liver metastases from uveal melanoma: a single-center experience. Ann Surg Oncol 2025;32:4989–4996 40246741 10.1245/s10434-025-17115-0 PMC12129856

[91] Hindso TG, Jensen PS, Sjol MB, Nissen K, Bjerrum CW, von Benzon E et al Impact of metastatic pattern on survival in patients with posterior uveal melanoma: a retrospective cohort study. Cancers (Basel) 2024;16:3346 39409966 10.3390/cancers16193346 PMC11475269

[92] Höppener DJ, Grünhagen DJ, Eggermont AMM, van der Veldt AAM, Verhoef C. An overview of liver directed locoregional therapies. Hematol Oncol Clin North Am 2025;39:103–123 39510668 10.1016/j.hoc.2024.08.010

[93] Rowcroft A, Loveday BPT, Thomson BNJ, Banting S, Knowles B. Systematic review of liver directed therapy for uveal melanoma hepatic metastases. HPB (Oxford) 2020;22:497–505 31791894 10.1016/j.hpb.2019.11.002

[94] Ahmed KA, Caudell JJ, El-Haddad G, Berglund AE, Welsh EA, Yue B et al Radiosensitivity differences between liver metastases based on primary histology suggest implications for clinical outcomes after stereotactic body radiation therapy. Int J Radiat Oncol Biol Phys 2016;95:1399–1404 27319288 10.1016/j.ijrobp.2016.03.050 PMC7771381

[95] Tran DH, Shanley R, Giubellino A, Tang PH, Koozekanani DD, Yuan J et al Radiation and systemic immunotherapy for metastatic uveal melanoma: a clinical retrospective review. Front Oncol 2024;14:1406872 39026970 10.3389/fonc.2024.1406872 PMC11254688

[96] Sajan A, Fordyce S, Sideris A, Liou C, Toor Z, Filtes J et al Minimally invasive treatment options for hepatic uveal melanoma metastases. Diagnostics (Basel) 2023;13:1836 37296688 10.3390/diagnostics13111836 PMC10253082

[97] Gonsalves CF, Adamo RD, Eschelman DJ. Locoregional therapies for the treatment of uveal melanoma hepatic metastases. Semin Intervent Radiol 2020;37:508–517 33328707 10.1055/s-0040-1720948 PMC7732575

[98] Sato T . Locoregional management of hepatic metastasis from primary uveal melanoma. Semin Oncol 2010;37:127–138 20494705 10.1053/j.seminoncol.2010.03.014

[99] Kennedy AS, Nutting C, Jakobs T, Cianni R, Notarianni E, Ofer A et al A first report of radioembolization for hepatic metastases from ocular melanoma. Cancer Invest 2009;27:682–690 19219675 10.1080/07357900802620893

[100] Gonsalves CF, Eschelman DJ, Adamo RD, Anne PR, Orloff MM, Terai M et al A prospective phase II trial of radioembolization for treatment of uveal melanoma hepatic metastasis. Radiology 2019;293:223–231 31453767 10.1148/radiol.2019190199 PMC6776232

[101] Huibers A, DePalo DK, Perez MC, Zager JS, Olofsson Bagge R. Isolated hyperthermic perfusions for cutaneous melanoma in-transit metastasis of the limb and uveal melanoma metastasis to the liver. Clin Exp Metastasis 2023;41:447–456 37843790 10.1007/s10585-023-10234-6 PMC11374821

[102] Olofsson Bagge R, Nelson A, Shafazand A, All-Eriksson C, Cahlin C, Elander N et al Isolated hepatic perfusion with melphalan for patients with isolated uveal melanoma liver metastases: a multicenter, randomized, open-label, phase III trial (the SCANDIUM trial). J Clin Oncol 2023;41:3042–3050 36940407 10.1200/JCO.22.01705 PMC10414734

[103] Olofsson Bagge R, Nelson A, Shafazand A, All-Eriksson C, Cahlin C, Elander N et al Survival and quality of life after isolated hepatic perfusion with melphalan as a treatment for uveal melanoma liver metastases—final results from the phase III randomized controlled trial SCANDIUM. Ann Surg 2024;282:100–107 38420778 10.1097/SLA.0000000000006255 PMC12140551

[104] Ku Y, Saitoh M, Nishiyama H, Fujiwara S, Iwasaki T, Ohyanagi H et al [Extracorporeal adriamycin-removal following hepatic artery infusion: use of direct hemoperfusion combined with veno-venous bypass]. Nihon Geka Gakkai Zasshi 1989;90:1758–1764 2594003

[105] Pingpank JF, Libutti SK, Chang R, Wood BJ, Neeman Z, Kam AW et al Phase I study of hepatic arterial melphalan infusion and hepatic venous hemofiltration using percutaneously placed catheters in patients with unresectable hepatic malignancies. J Clin Oncol 2005;23:3465–3474 15908655 10.1200/JCO.2005.00.927 PMC2374756

[106] Meijer TS, Burgmans MC, de Leede EM, de Geus-Oei LF, Boekestijn B, Handgraaf HJM et al Percutaneous hepatic perfusion with melphalan in patients with unresectable ocular melanoma metastases confined to the liver: a prospective phase II study. Ann Surg Oncol 2021;28:1130–1141 32761328 10.1245/s10434-020-08741-x PMC7801354

[107] Zager JS, Orloff M, Ferrucci PF, Choi J, Eschelman DJ, Glazer ES et al Efficacy and safety of the melphalan/hepatic delivery system in patients with unresectable metastatic uveal melanoma: results from an open-label, single-arm, multicenter phase 3 study. Ann Surg Oncol 2024;31:5340–5351 38704501 10.1245/s10434-024-15293-x PMC11249544

[108] Bethlehem MS, Katsarelias D, Olofsson Bagge R. Meta-analysis of isolated hepatic perfusion and percutaneous hepatic perfusion as a treatment for uveal melanoma liver metastases. Cancers (Basel) 2021;13:4726 34572953 10.3390/cancers13184726 PMC8469397

[109] Olofsson Bagge R, Nelson A, Shafazand A, Cahlin C, Carneiro A, Helgadottir H et al A phase Ib randomized multicenter trial of isolated hepatic perfusion in combination with ipilimumab and nivolumab for uveal melanoma metastases (SCANDIUM II trial). ESMO Open 2024;9:103623 38959698 10.1016/j.esmoop.2024.103623 PMC11269777

[110] Tong TML, Burgmans MC, Speetjens FM, van Erkel AR, van der Meer RW, van Rijswijk CSP et al Combining melphalan percutaneous hepatic perfusion with ipilimumab plus nivolumab in advanced uveal melanoma: first safety and efficacy data from the phase Ib part of the CHOPIN trial. Cardiovasc Intervent Radiol 2023;46:350–359 36624292 10.1007/s00270-022-03338-1

[111] Minor DR, Sato T, Orloff M, Luke J, Eschelman DJ, Gonsalves CF et al Initial report of treatment of uveal melanoma with hepatic metastases with yttrium90 internal radiation followed by ipilimumab and nivolumab. J Clin Oncol 2020; 38(Suppl): 10025

[112] Peuker C-AA, De Bucourt M, Gebauer B, Amthauer H, Erxleben C, Eucker J et al First interim analysis of the SirTac trial: a randomized phase II study of SIRT and DSM-TACE in patients with liver metastases from uveal melanoma [abstract]. J Clin Oncol 2022;40(Suppl. 16):9511

[113] Valsecchi ME, Terai M, Eschelman DJ, Gonsalves CF, Chervoneva I, Shields JA et al Double-blinded, randomized phase II study using embolization with or without granulocyte-macrophage colony-stimulating factor in uveal melanoma with hepatic metastases. J Vasc Interv Radiol 2015;26:523–532.e2 25678394 10.1016/j.jvir.2014.11.037 PMC4417549

[114] Ziemlewicz TJ, Critchfield JJ, Mendiratta-Lala M, Wiggermann P, Pech M, Serres-Créixams X et al The #HOPE4LIVER single-arm pivotal trial for histotripsy of primary and metastatic liver tumors: 1-year update of clinical outcomes. Ann Surg 2025; DOI: 10.1097/SLA.0000000000006720 [Epub ahead of print] PMC12594125 40201962

[115] Scheffer HJ, Nielsen K, de Jong MC, van Tilborg AA, Vieveen JM, Bouwman AR et al Irreversible electroporation for nonthermal tumor ablation in the clinical setting: a systematic review of safety and efficacy. J Vasc Interv Radiol 2014;25:997–1011; quiz 1011 24656178 10.1016/j.jvir.2014.01.028

[116] Sacco JJ, Harrington KJ, Olsson-Brown A, Chan TY, Nenclares P, Leslie I et al Safety, efficacy, and biomarker results from an open-label, multicenter, phase 1 study of RP2 alone or combined with nivolumab in a cohort of patients with uveal melanoma. J Clin Oncol 2024;42(Suppl):9511

[117] Beasley AB, Chen FK, Isaacs TW, Gray ES. Future perspectives of uveal melanoma blood based biomarkers. Br J Cancer 2022;126:1511–1528 35190695 10.1038/s41416-022-01723-8 PMC9130512

[118] Russo A, Caltabiano R, Longo A, Avitabile T, Franco LM, Bonfiglio V et al Increased levels of miRNA-146a in serum and histologic samples of patients with uveal melanoma. Front Pharmacol 2016;7:424 27895580 10.3389/fphar.2016.00424 PMC5108814

[119] Ragusa M, Barbagallo C, Statello L, Caltabiano R, Russo A, Puzzo L et al miRNA profiling in vitreous humor, vitreal exosomes and serum from uveal melanoma patients: pathological and diagnostic implications. Cancer Biol Ther 2015;16:1387–1396 25951497 10.1080/15384047.2015.1046021 PMC4622662

